# Establishment of dual reverse transcriptase-polymerase chain reaction for detection system for Areca palm velarivirus 1

**DOI:** 10.1371/journal.pone.0303941

**Published:** 2024-06-05

**Authors:** Chunlin Peng, Benyi Fan, Hui Zhu, Liyun Liu, Zhengwu Zhao, Liyun Huang

**Affiliations:** 1 Coconut Research Institute, Chinese Academy of Tropical Agricultural Sciences, Hainan, China; 2 College of Life Sciences, Chongqing Normal University, Chongqing, China; ICAR-Central Plantation Crops Research Institute, INDIA

## Abstract

Areca palm velarivirus 1 (APV1) is one of the main pathogen causing yellow leaf disease, and leading to considerable losses in the Areca palm industry. The detection methods for APV1 are primarily based on phenotype determination and molecular techniques, such as polymerase chain reaction (PCR). However, a single PCR has limitations in accuracy and sensitivity. Therefore, in the present study, we established a dual RT-PCR APV1-detection system with enhanced accuracy and sensitivity using two pairs of specific primers, YLDV2-F/YLDV2-R and YLDV4-F/YLDV4-R. Moreover, two cDNA fragments covering different regions of the viral genome were simultaneously amplified, with PCR amplicon of 311 and 499 bp, respectively. The dual RT-PCR detection system successfully amplified the two target regions of the APV1, demonstrating high specificity and sensitivity and compensating for the limitations of single-primer detection methods. We tested 60 Areca palm samples from different geographical regions, highlighting its advantages in that the dual RT-PCR system efficiently and accurately detected APV1 in samples across diverse areas. The dual RT-PCR APV1 detection system provides a rapid, accurate, and sensitive method for detecting the virus and offers valuable technical support for research in preventing and managing yellow leaf diseases caused by APV1 in Areca palms. Moreover, the findings of this study can serve as a reference for establishing similar plants viral detection systems in the future.

## Introduction

Areca palm (*Areca catechu L*.) is a medicinal plant widely cultivated in tropical and subtropical regions and holds high economic and medicinal value. Areca palm originated in Malaysia and the Philippines, and is extensively distributed in most of the tropical Pacific, Asia, and East Africa. In China, the Areca palm is primarily found in the Hainan Province and is a major economic source for farmers, accounting for approximately 95% of the national planting area. The Areca palm industry in Hainan has become the center of the Chinese Areca palm industry. The largest global arecanut producer is India; the Areca palm is primarily grown in regions such as Karnataka and has deep-rooted cultural significance as a traditional crop. Owing to its importance, the Areca palm industry has become a major source of income for Indian farmers [1–5].

Yellow leaf disease (YLD) severely threatens Areca palm plantations worldwide. The disease was first discovered in Indian Areca palm trees in 1914 [6,7] and subsequently reported in China in 1981 [8] and Sri Lanka in 2015 reported [9], YLD, causes extensive damage to Areca palm crops, severely threatening plantations worldwide. Areca palm velarivirus 1 (APV1) is one of the main pathogens causing YLD in Areca palms, which is widely spread in areca palm plantationsand difficult to control, APV1 is a novel member of the genus *Velarivirus* within the family *Closteroviridae*. APV1 exhibits typical flexuous, filamentous particles and possesses a positive-sense, single-stranded RNA genome of 17,546 nucleotides (nt), encoding 11 open reading frames, APV1 is the sole velarivirus known to be trans-mitted by mealybugs [10,13]. Symptoms of the disease first appear on the tips or middle crowns of the lower leaves, which exhibit distinct abnormal yellowing and normal green areas. The yellowing expands toward the vascular tissue, whereas the midrib remains green, forming a yellow-green boundary, which distinguishes it from physiological yellowing. Subsequently, the margins of the older leaves turn yellow-brown, and the loss of green color extends to the newly emerging tender leaves. In the later stages, the infected leaves developed poorly and the width of the crown significantly reduces, leading to the “top-bunching” symptom. As the disease progresses, the rhizomes rot and fruit size decreases, stems become soft and brittle, and the top eventually breaks off [7,11–13]. Currently, there is no effective control method for YLD, and breeding resistant varieties remains the standard approach for preventing and controlling the disease in Areca palm cultivation.

The detection methods for YLD are diverse, including phenotypice and molecular methods. The physiological methods primarily involve observing the growth and development of infected plants, leaf morphology, and changes in leaf color to make judgments. Although this method is simple and easy to implement, it has certain limitations regarding the identification accuracy and sensitivity [14]. Molecular methods are currently the most advanced and accurate identification techniques. These methods mainly utilize polymerase chain reaction (PCR), real-time polymerase chain reaction (real-time PCR), and immunocapture RT-PCR [5,15,16]. Among these, PCR is the most cost-effective and commonly used for viral detection in experimental procedures.

However, in this study, a single RT-PCR was modified to by target the two regions in the viral genome simultaneously using two sets of viral primers in a single sample, establishing a dual RT-PCR detection system. This approach reduced the probability of false positives, increased the detection accuracy, and eliminated the need for repeated testing to confirm infection status. Compared to single RT-PCR, double RT-PCR achieved two experimental results with a single amplification, thereby improving the detection efficiency, reducing the detection cost, overcoming the limitations of single-primer detection, and enhancing the reliability of the results. Double RT-PCR can ensure the rapid, efficient, and accurate detection of APV1 in experiments. However, to our knowledge, there have been no reports published on this method. Thus, the present study can enhance YLD detection in Areca palm.

## Materials and methods

### Experimental materials

In the present study, we collected 60 samples from diseased palm plantations in Wanning (longitude and latitude: 110.388793, 18.796216) and Qionghai (longitude and latitude: 110.409471, 19.243028). During sampling, two or three yellow-green alternating leaves from the middle of each Areca palm plant were selected. The collected samples were rapidly placed in pre-labeled sterile sampling bags. Healthy samples without yellowing symptoms were collected for the mixed testing. The RNA was extracted in the laboratory on the same day as the sampling to ensure the sample quality. Samples that were not processed immediately were promptly stored at −80°C to prevent excessive degradation of leaf RNA.

## Major reagents and instruments

The RNAprep pure polysaccharide plant total RNA extraction kit, One-Step Genomic DNA Removal and cDNA Synthesis PreMix, and 2×Taq PCR PreMix II (KT211-02 with dye) were purchased from TianGen Biotech Co., Ltd. (Beijing, China). The RT-PCR primers were synthesized by Shanghai Sangon Biotech Co., Ltd. Mercaptoethanol, GelStain nucleic acid dye, chloroform, and 50×TAE were used in the experiment. The main instruments used were a Jena TOne PCR instrument (Jena Analytical Instruments Co., Ltd., Germany), a high-speed centrifuge (Henan North Hong Industrial Co., Ltd.), an electrophoresis apparatus (Beijing Liuyi Biological Technology Co., Ltd.), a high-speed, low-temperature tissue grinder (Wuhan Saivi Biotechnology Co., Ltd.), and a G: BOX F3 gel imaging system (Syngene of Synoptics Group, UK).

### Total RNA extraction and reverse transcription

Approximately 0.05 g of fresh Areca palm leaves with yellow-green alternating colors were cut and slowly transferred into enzyme-free, sterile centrifuge tubes. The samples were quickly placed in liquid nitrogen for preservation. Once all the samples were cut, they were uniformly placed in a high-speed, low-temperature tissue grinder (Wuhan Saiweier Biotechnology Co., Ltd) and mixed with three steel beads with a diameter of 4 mm for grinding. Subsequently, the total RNA was extracted from the Areca palm plants following the RNAprep Pure Polysaccharide Polyphenol Plant Total RNA Extraction Kit instructions (Tiangen Biochemical Technology (Beijing) Co., Ltd). After the total RNA extraction, the samples were placed on ice for reverse transcription to reduce the risk of RNA degradation and ensure data accuracy.

### Single RT-PCR amplification

cDNA templates were synthesized using a One-Step Genomic DNA Removal and cDNA Synthesis PreMix (Tiangen Biochemical Technology (Beijing) Co., Ltd). Both reagents and total RNA were stored in an icebox. 5×FastKing-RT Super Mix (4 μL) and total RNA (2 μL) were added to a sterile enzyme-free PCR tube. An appropriate amount of RNase-free ddH_2_O was added to ensure a total volume of 20 μL. After brief centrifugation, the mixture was placed into a PCR instrument and subjected to a 15-min reaction at 42°C to synthesize cDNA and remove genomic DNA and a 3-min reaction at 95°C to inactivate the enzymes. The synthesized cDNA was stored at −20°C until ready for further use.

The reaction system volume for the single RT-PCR was 25 μL, consisting of 1 μL of forward primer (10 μmol/L), 1 μL of reverse primer (10 μmol/L), 12.5 μL of 2×Taq PCR PreMix II (KT211-02 with dye) (Tiangen Biochemical Technology (Beijing) Co., Ltd), 2 μL of DNA template, and 8.5 μL of ddH_2_O. The PCR cycling parameters were as follows: pre-denaturation at 95°C for 4 min, denaturation at 95°C for 30 s, annealing at 55°C for 30 s, extension at 72.0°C for 1 min, 35 cycles of amplification, and a final extension at 72.0°C for 10 min. The RT-PCR products were stained with GelStain nucleic acid dye and analyzed via 1% agarose gel electrophoresis.

### Establishment of dual RT-PCR amplification system

#### Optimization of dual RT-PCR reaction conditions

Optimization of the dual RT-PCR reaction conditions is crucial to obtain highly specific and sensitive detection results. The annealing temperature was set at 45, 50, 55, and 60°C, and the cDNA template volume was varied using five different system volumes: 0.5, 1, 1.5, 2, and 2.5 μL. The primer volumes were adjusted using five different systems: 0.4, 0.6, 0.8, 1, and 1.2 μL. The most suitable dual RT-PCR reaction conditions were determined by systematically optimizing the above parameters. The process considered the repeatability and stability of the reaction results to ensure the generation of reliable experimental data. The goal was to develop an optimized dual RT-PCR system that provides accurate and reproducible outcomes with high specificity and sensitivity for target detection.

### Optimization of dual RT-PCR reaction system and reaction protocol

Two pairs of dual RT-PCR primers, YLDV2-F/YLDV2-R (position 8325–8635 nt) and YLDV4-F/YLDV4-R (9901–10400 nt) [5] (Table 1), were added in a 1:1 volume ratio.

**Table 1:** PCR amplification primers used in the study.

Primer	Primer sequence (5′–3′)	Primer length(bp)	Size of characteristic band (bp)
YLDV2–F YLDV2–R YLDV4–F YLDV4–R	GATCTGTGAATATATCAGAACA CACCCTTGGTATCAACAATAGA ATCTGGACCGAGTAATGGGA TCATTGTGATACACATACAAGT	22 22 20 22	311 499

The dual RT-PCR system consisted of 2 μL of template, 1 μL (10 μmol/L) of each pair of upstream and downstream primers, and 12.5 μL of 2×Taq PCR PreMix II (KT211-02 with dye), and RNase-free ddH_2_O was added to bring the total volume to 25 μL. The optimal RT-PCR conditions were determined by changing the reaction conditions. The dual RT-PCR protocol was similar to that of the single RT-PCR protocol.

### Sensitivity determination of dual RT-PCR

Total cDNA containing APV1 was diluted in 10-fold concentration gradients (10^−0^, 10^−1^, 10^−2^, 10^−3^, 10^−4^, and 10^−5^). The diluted cDNA was used as a template, and both the single RT-PCR and optimized dual RT-PCR systems were employed for detection. The PCR products were subjected to 1% agarose gel electrophoresis, and the sensitivities of the single RT-PCR and optimized dual RT-PCR were compared.

### Sequence alignment

After verification via 1.0% agarose gel electrophoresis and imaging of the single RT-PCR amplification products, 15 APV1-positive samples detected using each pair of primers were randomly selected and sent to the Guangzhou Bioengineering for sequencing. The obtained APV1 cDNA target sequences were assembled using DNAMAN software, and the sequences were subjected to BLAST analysis and visualization on NCBI (https://www.ncbi.nlm.nih.gov/) for comparison.

## Results

### Single PCR detection

**Fig 1:**
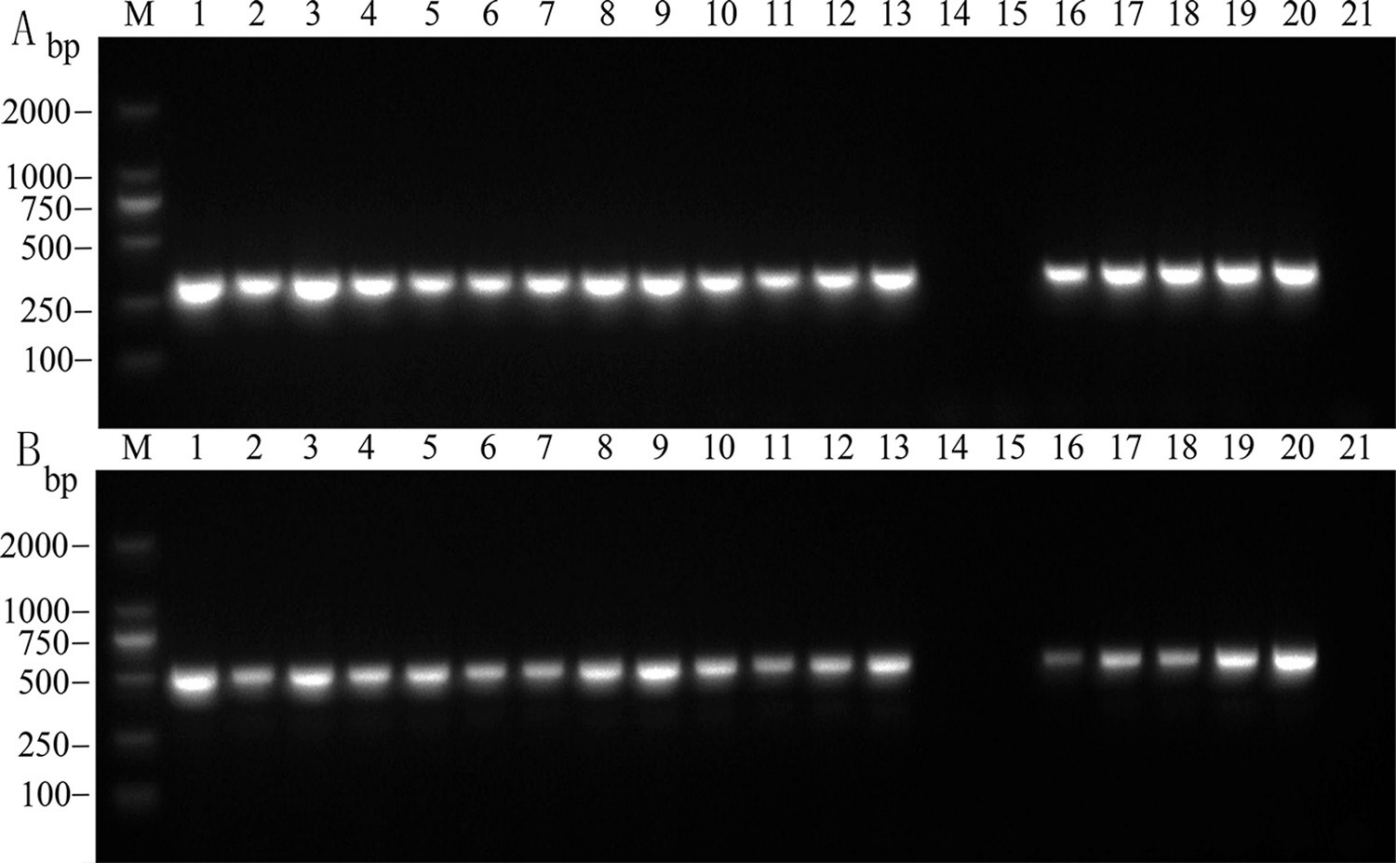
Single PCR detection results using primers. A: YLDV2-F/YLDV2-R. M: DL2000 DNA marker; 1−20: Areca palm samples; 21: Blank control. B: YLDV4-F/YLDV4-R. M: DL2000 DNA marker; 1−20: Areca palm samples; 21: Blank control.

To detect YLD caused by APV1 in arecanuts, we used two pairs of RT-PCR primers sets to target two regions in the APV1 genome. The resulted RT-PCR amplicons were 311 and 499 bp (Fig 1A and 1B), respectively. During the amplification process, we observed clear bands without any nonspecific bands, confirming the high specificity of the primers and indicating that these two primer pairs could be used in a dual RT-PCR detection system for APV1 infected Areca palm samples.

### Optimization of dual RT-PCR amplification system

#### Impact of annealing temperature on dual RT-PCR

**Fig 2:**
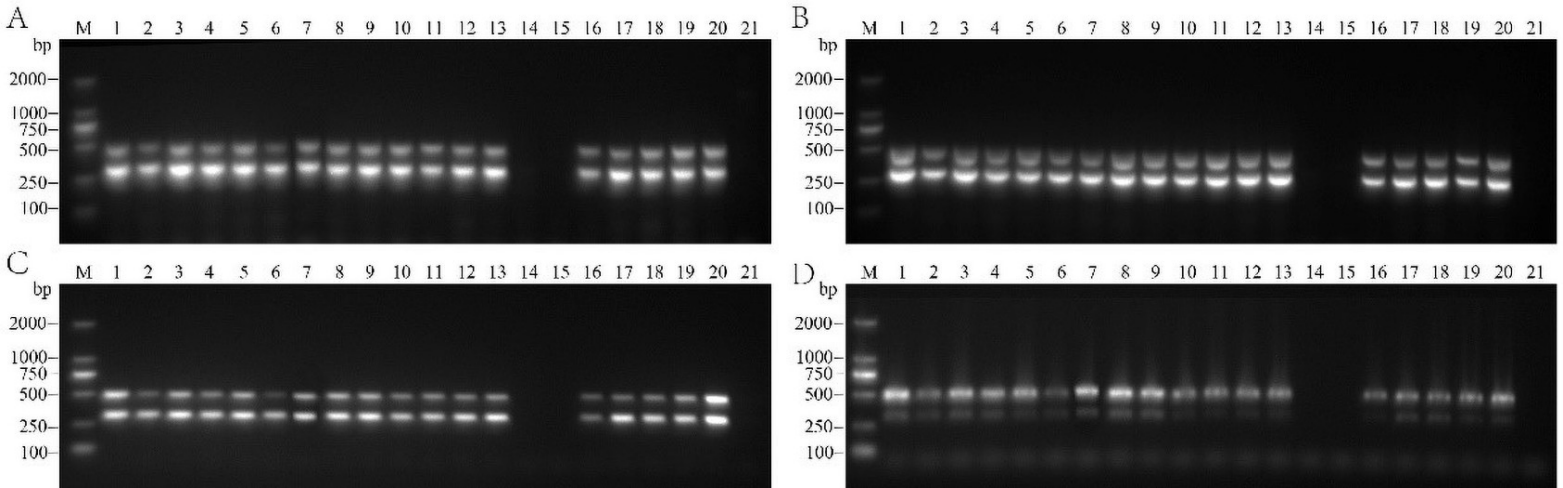
Effect of different annealing temperatures (A: 45°C; B: 50°C; C: 55°C; D: 60°C) on dual RT-PCR detection results. M: DL2000 DNA marker; 1−20: Areca palm samples; 21: Blank control.

At an annealing temperature of 45°C, the primer combinations YLDV2-F/YLDV2-R and YLDV4-F/YLDV4-R showed nonspecific amplification in the reaction system (Fig 2A). However, when the annealing temperature was increased to 50°C, the target bands of the samples were still amplified in the reaction system, although the clarity slightly decreased (Fig 2B). Further adjustment of the annealing temperature to 55°C eliminated nonspecific amplification in the reaction systems of both primer combinations (YLDV2-F/YLDV2-R and YLDV4-F/YLDV4-R). The amplified bands became single and bright with almost no nonspecific bands. The band intensity of the two target fragments was similar, indicating suitable conditions for dual-PCR amplification (Fig 2C). However, when the annealing temperature further increased to 60°C, the amplification bands of both primer pairs showed significant deformation, trailing, and a decreased primer binding ability, reducing the quality of the gel electrophoresis bands (Fig 2D).

These results indicate that the annealing temperature significantly impacted the specificity and efficiency of PCR. Adjusting the annealing temperature to 55°C resulted in high-quality PCR amplification with high clarity and strong specificity, making it highly suitable for the dual-PCR detection system of Areca palm samples.

#### Optimization of primer concentrations and cDNA amounts

**Fig 3:**
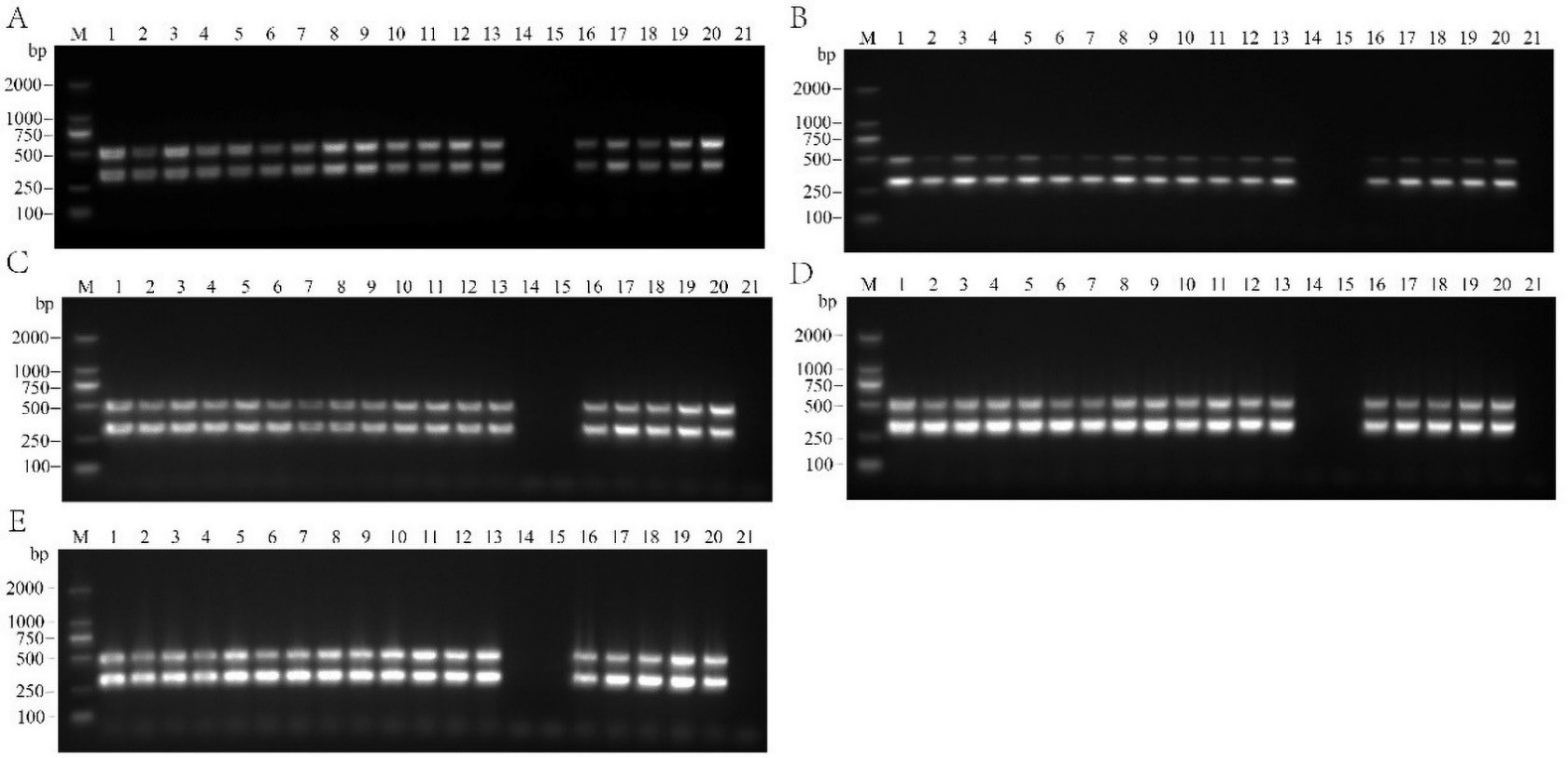
Effect of different reaction systems (A: System 1, B: System 2, C: System 3, D: System 4, E: System 5) on the detection results of dual RT-PCR. M: DL2000 DNA marker; 1−20: Areca palm samples; 21: Blank control.

To optimize the primer concentrations and cDNA amounts, we selected an annealing temperature of 55°C. Five reaction systems successfully amplified the corresponding target bands (Fig 3A–3E). After comparing and analyzing the experimental results, we finally selected reaction system 4 (Fig 3D) for APV1 dual RT-PCR detection. The amplification system was 25 μL in total, comprising 1 μL YLDV2-F (10 μmol/L), 1 μL YLDV2-R (10 μmol/L), 1 μL YLDV4-F (10 μmol/L), 1 μL YLDV4-R (10 μmol/L), 12.5 μL 2×Taq PCR PreMix II (KT211-02 with dye) (Tiangen Biochemical Technology (Beijing) Co., Ltd), 2 μL DNA template, and 6.5 μL ddH_2_O. The amplification program consisted of a pre-denaturation step at 95°C for 4 min, followed by denaturation at 95°C for 30 s, annealing at 55°C for 30 s, and extension at 72°C for 1 min, for a total of 35 cycles. The final extension was performed at 72°C for 10 min. Through a series of optimizations, we ensured the stability and specificity of the dual RT-PCR reaction, enabling accurate amplification of the target fragment. This reliable and efficient experimental protocol provided a robust method for detecting APV1.

#### Specificity detection

Single and dual RT-PCR amplification using the cDNA mixture as the template indicated that the two pairs of APV1 primers successfully amplified a single clear target band. Two pairs of primers also amplified 311 bp and 499 bp. In contrast, the dual RT-PCR system amplified two specific target bands, and the band positions were well distinguished, making it easy to differentiate between the two products.

#### Dual RT-PCR sensitivity detection

**Fig 4:**
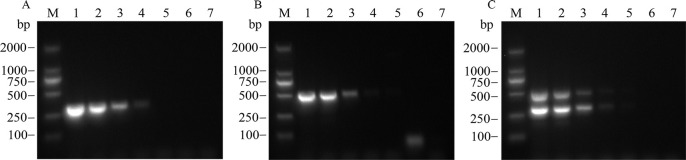
Sensitivity test for single RT-PCR and dual RT-PCR (A: YLDV2-F/YLDV2-R, B: YLDV4-F/YLDV4-R, C: Dual RT-PCR). M: DL2000 DNA marker; 1−6: cDNA diluted to 10^−0^, 10^−1^, 10^−2^,10^−3^, 10^−4^, and 10^−5^; 7: Blank control.

The cDNA template containing APV1 was diluted in 10-fold gradients and subjected to single and dual RT-PCR detections. The results showed that the lowest detectable cDNA concentration for single PCR was 10^−4^ using YLDV2-F/YLDV2-R (Fig 4A) but 10^−3^ using YLDV4-F/YLDV4-R (Fig 4B). However, for dual RT-PCR, the detectable cDNA concentration reached 10^−4^ (Fig 4C). Overall, the sensitivity of the dual RT-PCR assay was comparable to that of the single RT-PCR assay.

#### Sequence alignment results

**Fig 5:**
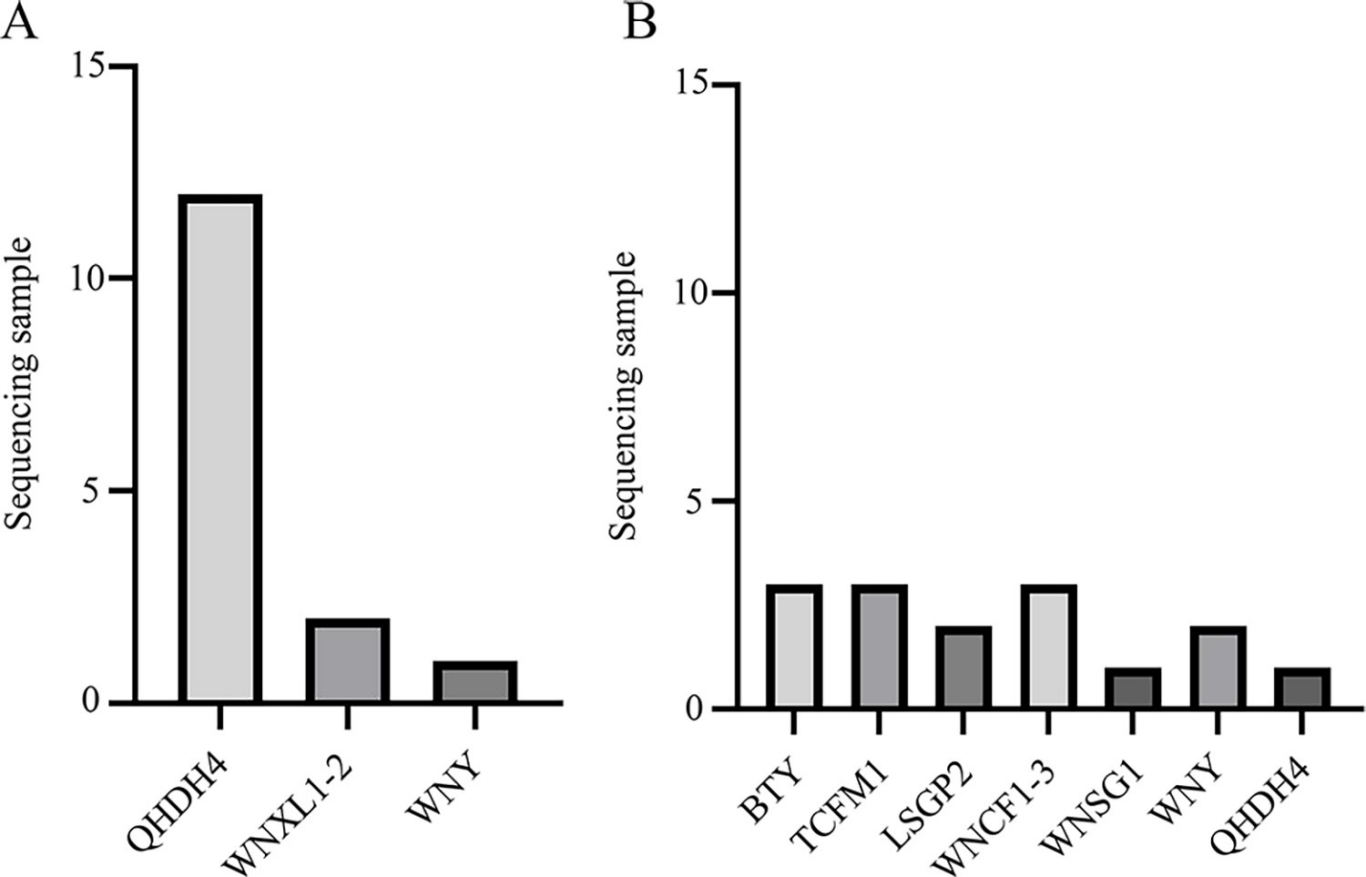
APV1 genome alignment results. (A: YLDV2-F/YLDV2-R sequence alignment results; B: YLDV4-F/YLDV4-R sequence alignment results).

To further confirm the accuracy of the detection results, we conducted sequence determination and homology comparisons of the PCR amplification products from 15 randomly selected positive samples for each pair of primers. All 30 samples amplified using YLDV2-F/YLDV2-R and YLDV4-F/YLDV4-R primers were positive for APV1 (Fig 5A and 5B). Among the samples amplified with the YLDV2-F/YLDV2-R primers, the sequences of sample1–sample12 showed 100% identity with the full genome RNA sequence of the APV1 QHDH4 (GenBank Acc. NO: MW316010), sample13–sample14 showed 100% identity with the APV WNXL–2 strain (GenBank Acc. NO: MW316022), and sample15 showed 100% identity with the APV–WNY (GenBank Acc. NO: MK956940).

Among the 15 samples amplified with YLDV4-F/YLDV4-R primers, sample1–sample3 showed 99% identity with the BTY (GenBank Acc. NO: MW316018), sample4–sample6 showed 99% identity with the TCFM1 (GenBank Acc. NO: MW316013), sample7–sample8 showed 100% identity with the LSGP2 (GenBank Acc. NO: MW316008), sample9–sample11 showed 99% identity with the WNCF1-3 (GenBank Acc. NO: MW316024), sample12 showed 100% identity with WNSG1 (GenBank Acc. NO: MW316016), sample13–sample14 showed 100% identity with APV-WNY (GenBank Acc. NO: MK956940), andsample15 showed 100% identity with QHDH4 (GenBank Acc. NO: MW316010). All alignment results confirmed that all sequenced samples that tested positive for APV1 were infected with APV1.

### Field detection of dual RT-PCR

**Fig 6:**
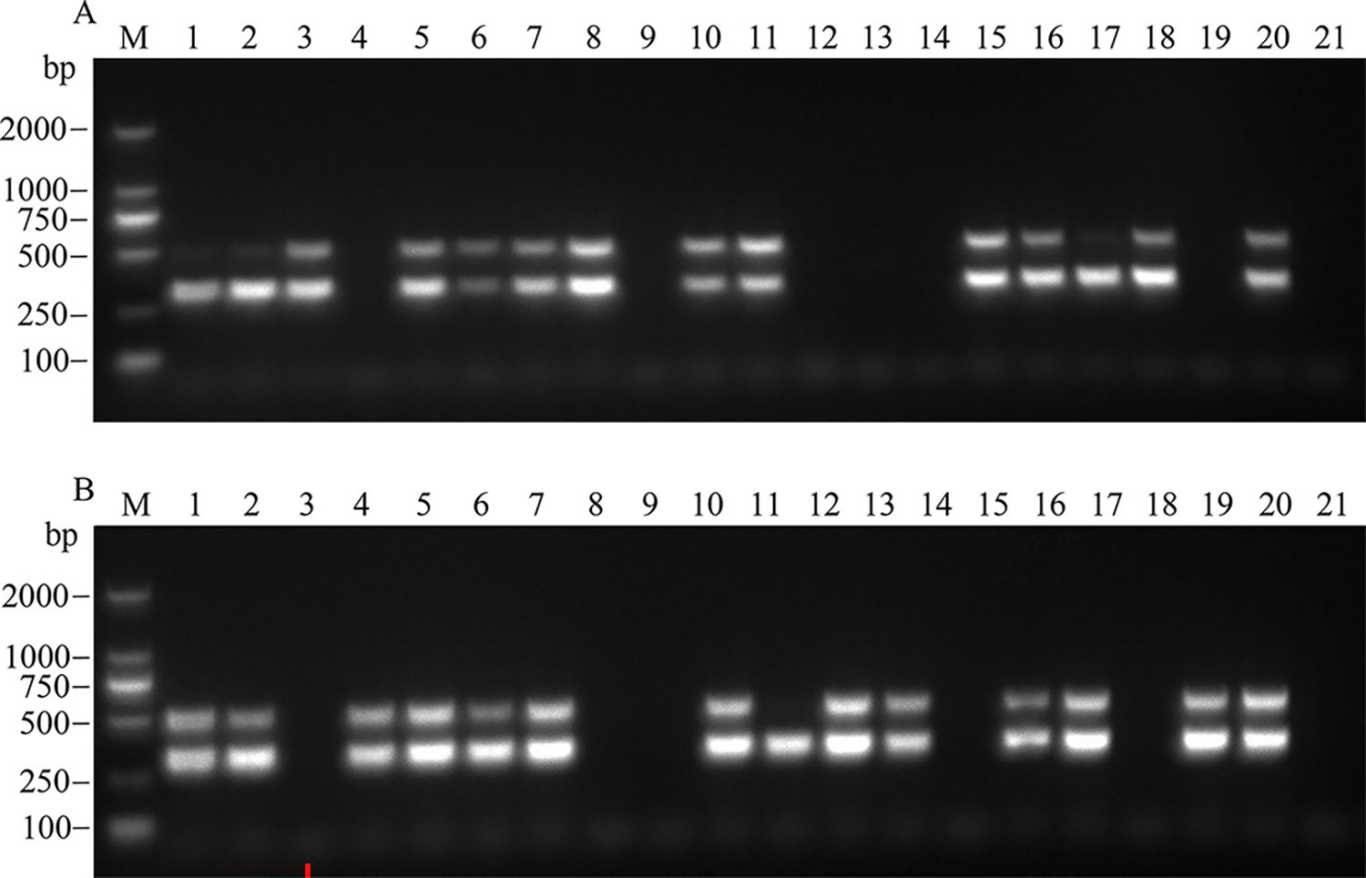
Results of dual RT-PCR detection of APV1 in some field-collected samples (A:1–20, B:1–20). M: DL2000 DNA marker; 1−20: Field-collected Areca palm samples; 21: Blank control.

We used a dual RT-PCR detection system to perform mixed detection on 60 samples collected from yellowing-diseased and non-diseased Areca palm plantations in Wanning and Qionghai. Most samples (using YLDV2-F/YLDV2-R and YLDV4-F/YLDV4-R primers) showed two clear bands corresponding to APV1 (Fig 6A and 6B). For samples that showed a single band in the system (based on experimental requirements), the RT-PCR products were sent to a biotech company for sequencing to ensure the accuracy of the detection results. This approach helped overcome these limitations and enhance the accuracy of single RT-PCR detection, improving the accuracy of the results and providing accurate detection results for YLD field management in Areca palms. This approach also enabled the timely treatment or felling of affected trees to prevent the spread of the virus and protect other Areca palm trees. The field detection results using dual RT-PCR were consistent with those obtained using the single RT-PCR, confirming that the established dual RT-PCR detection technology could be used for APV1 detection in Areca palm field samples.

## Discussion

In this study, we developed a dual RT-PCR detection method for APV1, which shows promising results for plant pathogen detection. Using specific PCR techniques and experimental procedures, we successfully established a rapid, accurate, and sensitive dual RT-PCR detection system targeting the viral genome. This method generates two distinct and clear bands in a single reaction, allowing positive samples to be unequivocally identified while reducing the difficulty of interpreting the results caused by weak bands or nonspecific amplification in practical operations. This approach enhances the accuracy of detection, overcomes the limitations of single RT-PCR, and demonstrates superiority in APV1 detection.

Multiple RT-PCR is an advanced method based on RT-PCR technology that allows for the simultaneous detection of multiple viruses or the use of multiple primer pairs to detect one virus. Offering comparable experimental steps and sensitivity to conventional RT-PCR, multiple RT-PCR reduces the time and cost associated with repetitive testing, reducing overall detection expenses. Consequently, this technique has been widely used to detect various plant viral diseases. For example, Hu et al. [17] employed dual RT-PCR to rapidly detect the apple stem-pitting virus. Zou et al. [18] detected three soil-borne pathogens, *Ralstonia solanacearum*, *Fusarium oxysporum* f. sp. *lycopersici*, and *Verticillium dahliae*, in diseased eggplant plants and soil using triple PCR with specific primers established for each pathogen. Moreover, Zou et al. [19] developed a multiplex RT-PCR method to simultaneously detect six potato viruses, one potato viroid, and one internal reference gene in a single reaction system through primer design and system optimization. Zhang et al. [20] established a multiplex RT-PCR system for simultaneously detecting five chrysanthemum viruses/classical viruses and optimized factors, such as primer ratios, annealing temperature, and template concentration, in the reaction system. Similar multiplex RT-PCR detection systems have also been established for other crops, such as tomato (*Solanum lycopersicum* L.), citrus (*Citrus reticulata* Blanco), sugarcane (*Saccharum officinarum* L.), garlic, and strawberries [21–25].

Multiple RT-PCR detection systems require careful primer screening/design, system design, and optimization to achieve optimal amplification results [26]. In the present study, the annealing temperature and reaction conditions were optimized. When the annealing temperature was too high or low, the amplification results were unsatisfactory. Improper template and primer concentration ratios also led to either the failure of target band amplification or the generation of nonspecific amplification products, consistent with the findings of Dong et al. [27] in their quadruple RT-PCR study. Through the optimization of annealing temperature, reaction systems, and other conditions, we determined the optimal reaction system for APV1 detection.

## Conclusions

This study optimized the dual RT-PCR system, enabling rapid and accurate detection of APV1 in samples across different regions, thereby fully leveraging the advantages of dual RT-PCR detection. The establishment of this detection system facilitates the rapid, accurate, and sensitive detection of APV1, providing crucial technical support for the prevention and control of yellowing diseases caused by APV1.

## Supporting information

S1 Raw images (PDF)

S1 Data (XLSX)
