# Supplementary material for: Establishment of dual reverse transcriptase-polymerase chain reaction for detection system for Areca palm velarivirus 1
Source: PLoS One. 2024 Jun 5;19(6):e0303941. doi: 10.1371/journal.pone.0303941 (PMC11152278; doi:10.1371/journal.pone.0303941)
Supplement: S1 Raw images — (PDF) [file pone.0303941.s001.pdf]

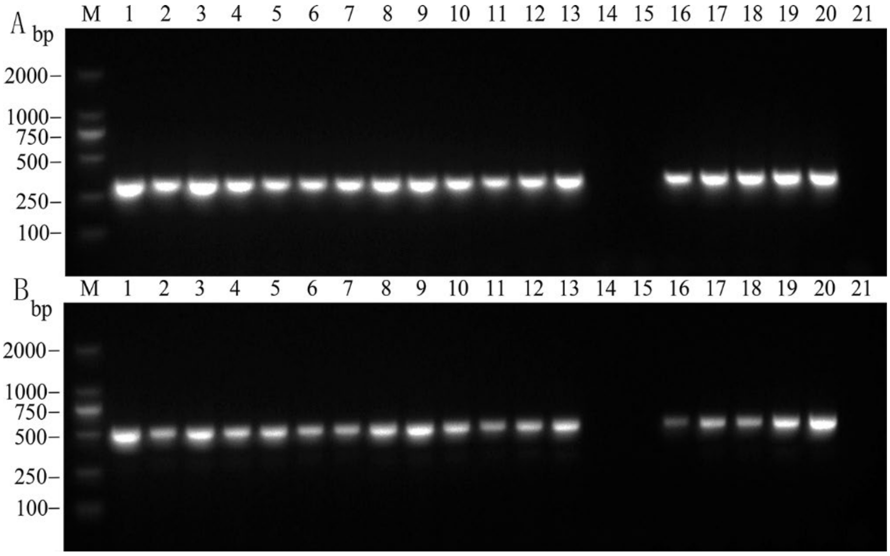
A bp
M 1 2 3 4 5 6 7 8 9 10 11 12 13 14 15 16 17 18 19 20 21
2000
1000
750
500
250
100
B bp
M 1 2 3 4 5 6 7 8 9 10 11 12 13 14 15 16 17 18 19 20 21
2000
1000
750
500
250
100

Fig 1

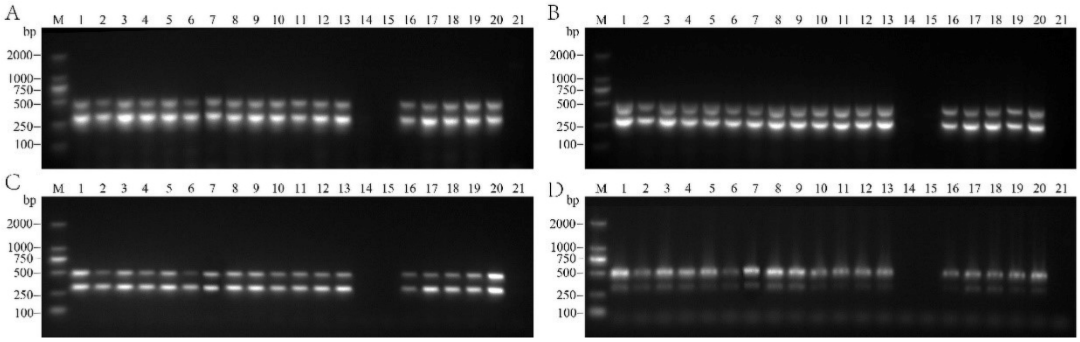
A
B
C
D

Fig 2

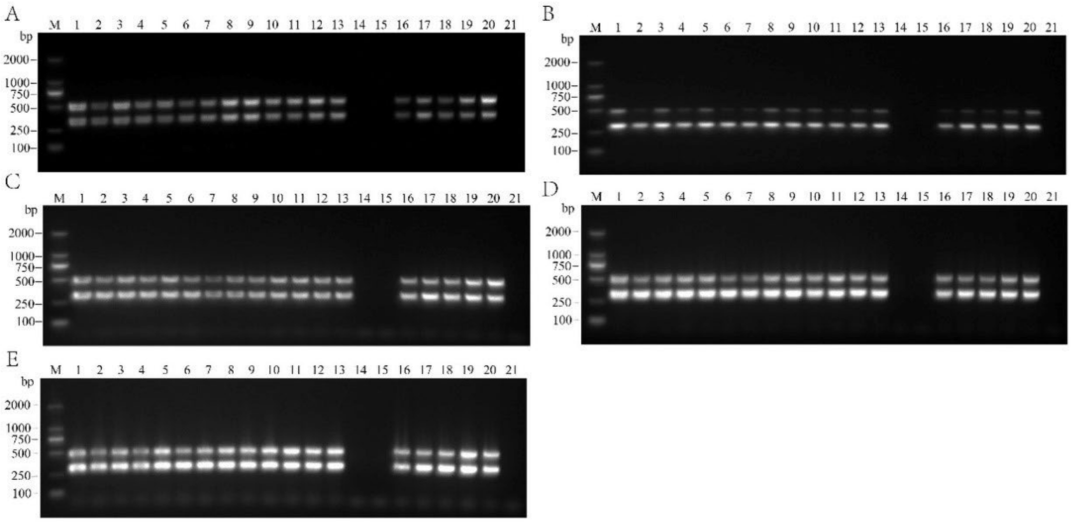

Fig 3

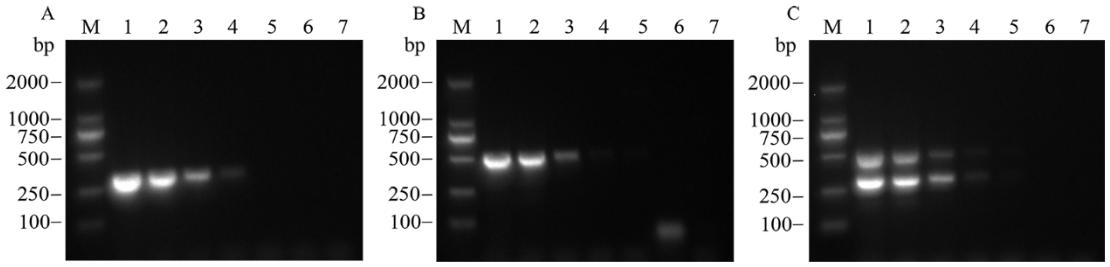

Fig4

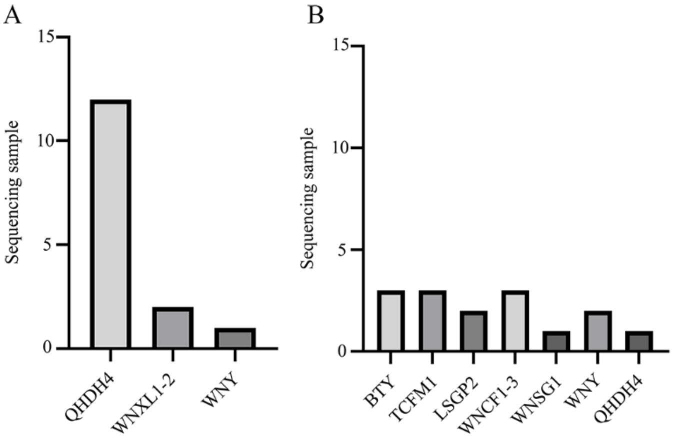

Fig 5

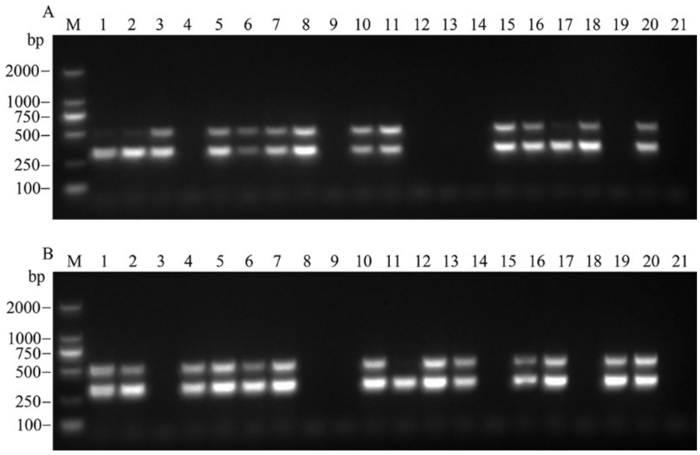
A
M 1 2 3 4 5 6 7 8 9 10 11 12 13 14 15 16 17 18 19 20 21
bp
2000
1000
750
500
250
100
B
M 1 2 3 4 5 6 7 8 9 10 11 12 13 14 15 16 17 18 19 20 21
bp
2000
1000
750
500
250
100

Fig 6

Original images for blots and gels

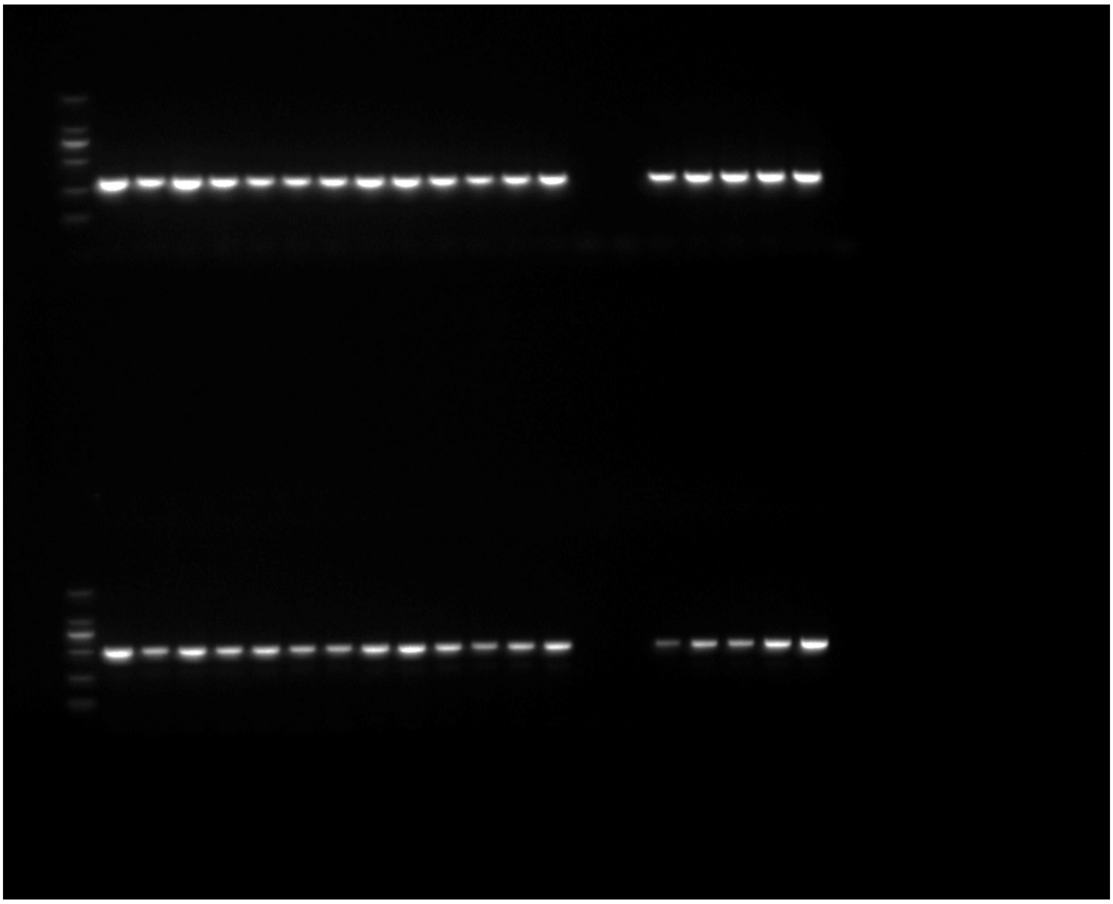

Single PCR detection results using primers(A-B)

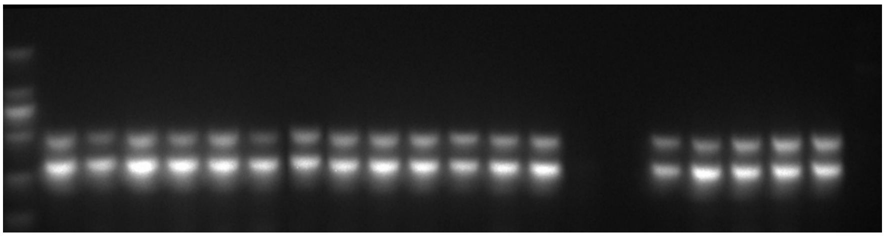

45 ℃ Dual RT-PCR(A)

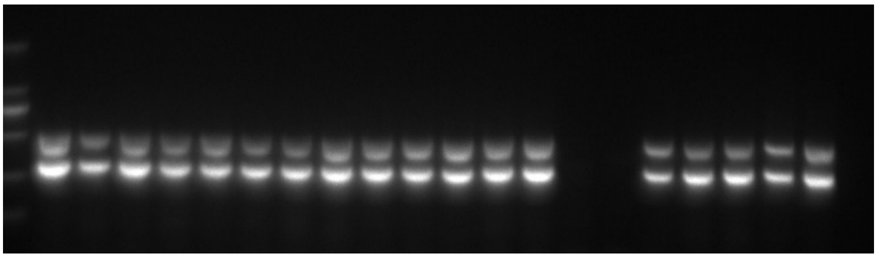

50 ℃ Dual RT-PCR(B)

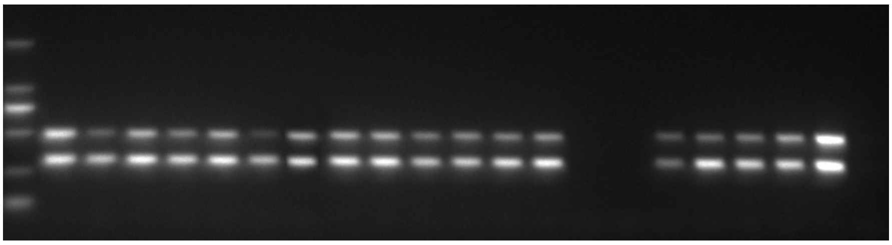

55 ℃ Dual RT-PCR(C)

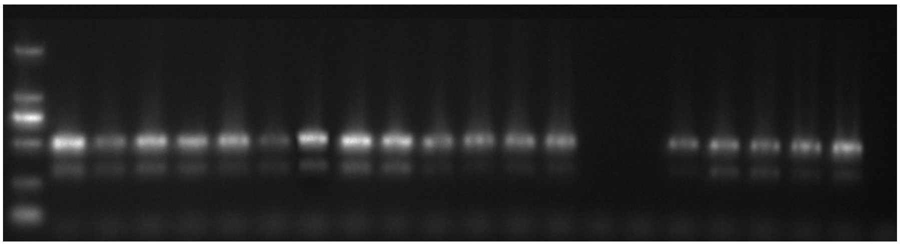

60 ℃ Dual RT-PCR(D)

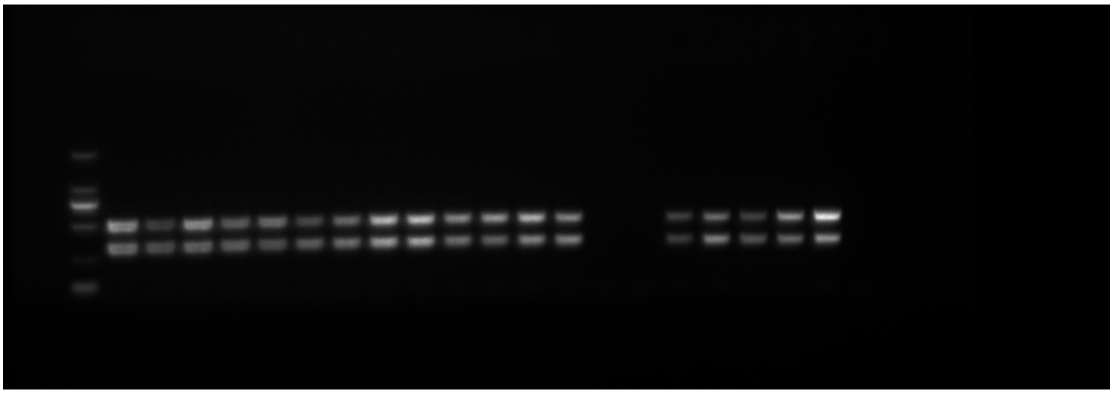

system 1(A)

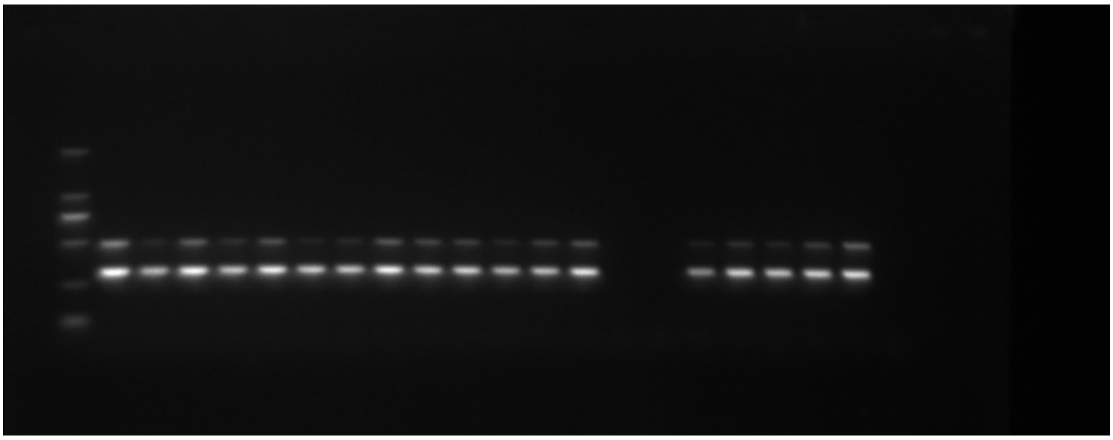

system 2(B)

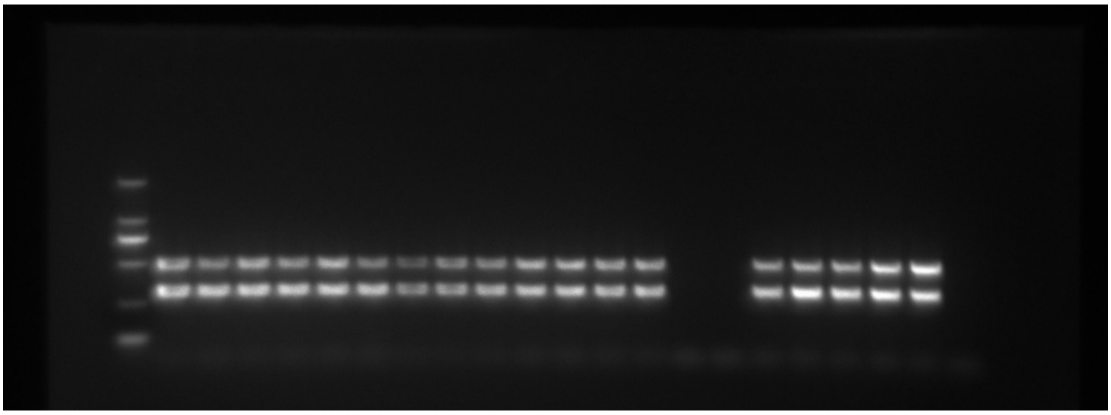

system 3(C)

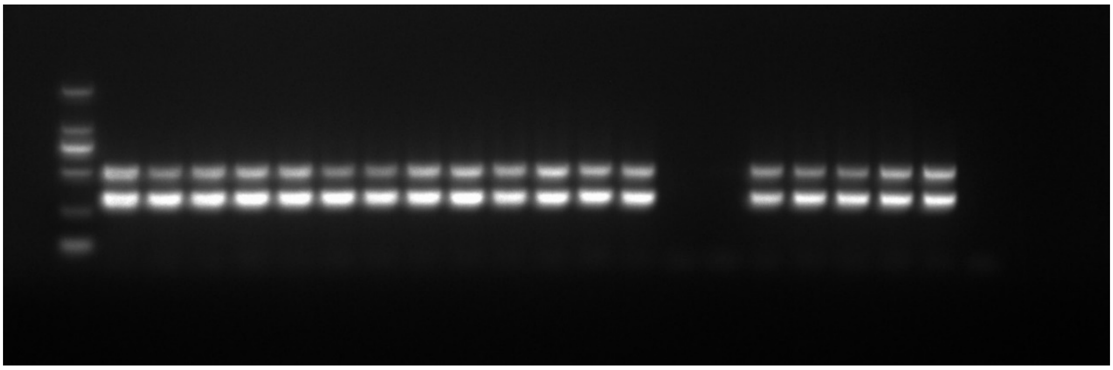

system 4(D)

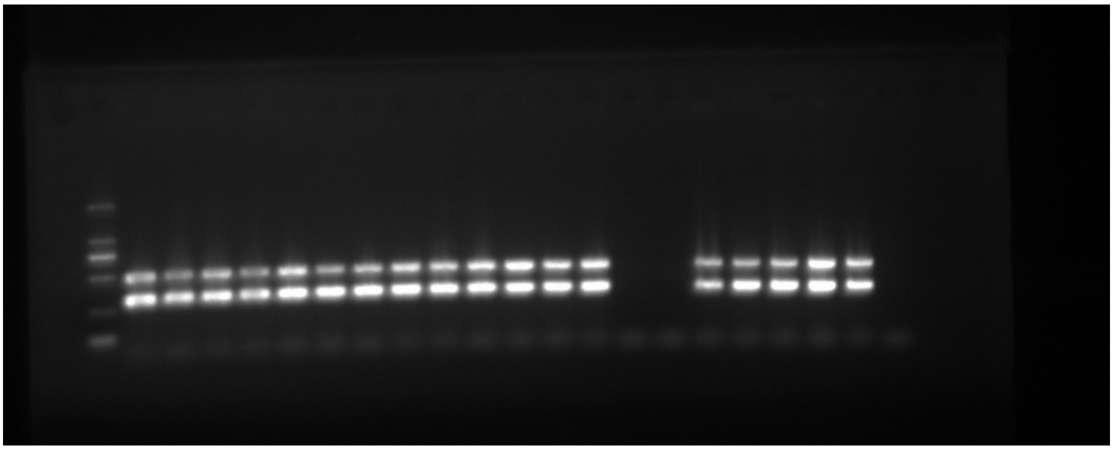

system 5(E)

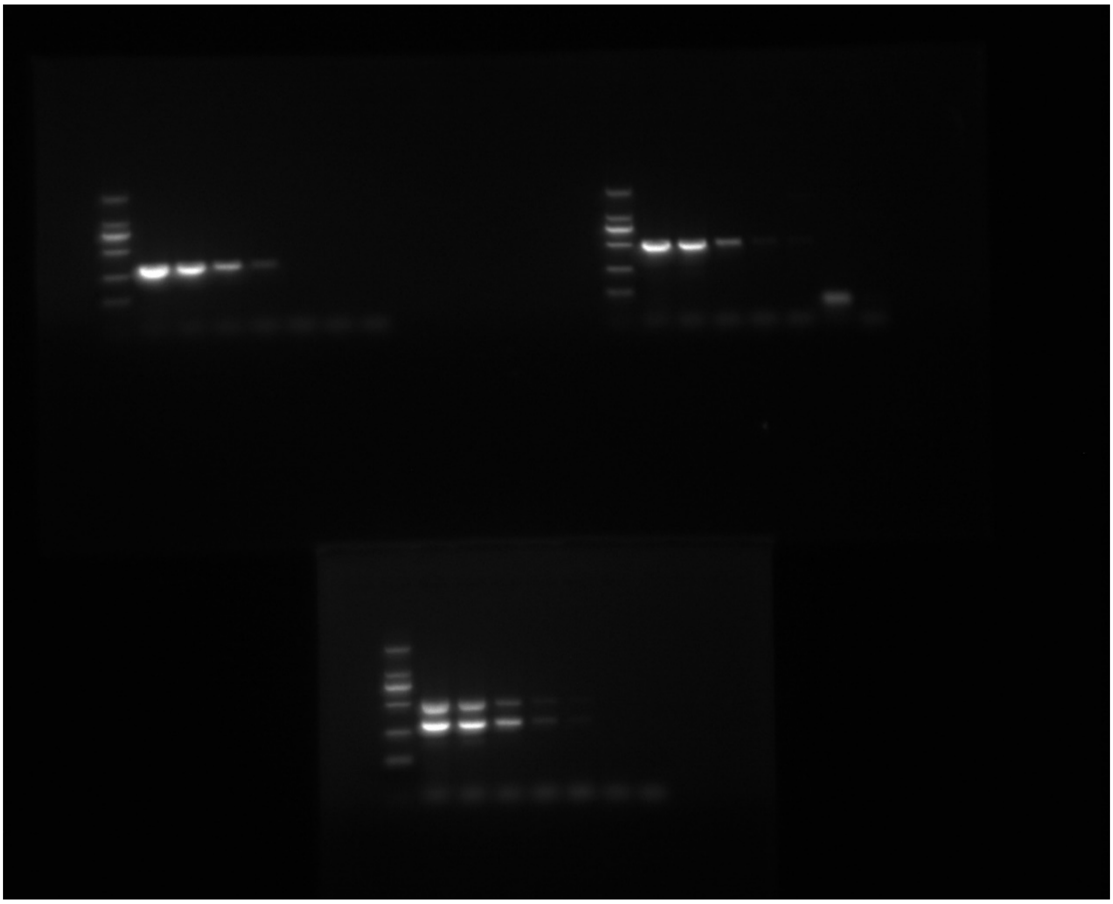

cDNA diluted to(A) (B) (C)

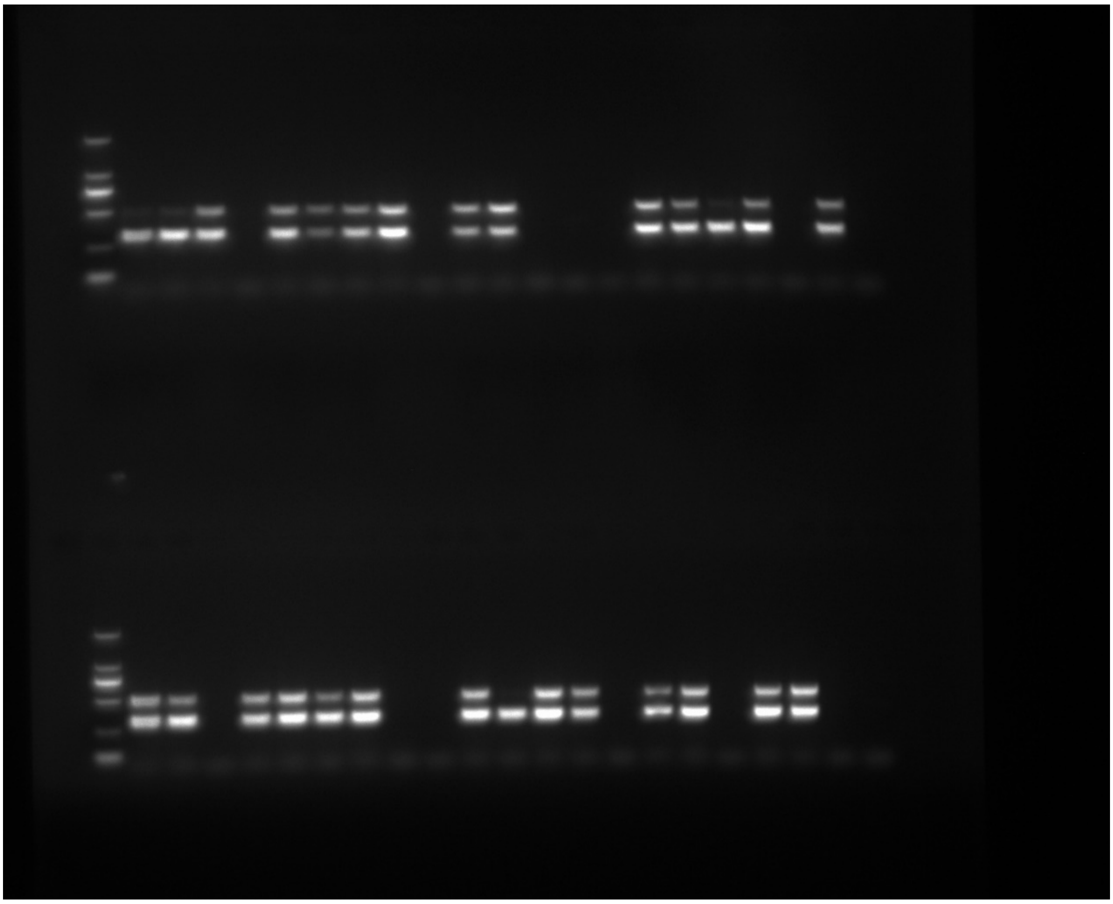

Results of dual RT-PCR detection of APV1 in some field collected samples(A) (B)
